# Emergency Services Utilization and Hospital Bed Management in Costa Rica: A Review

**DOI:** 10.4236/ojapps.2025.153047

**Published:** 2025-03-25

**Authors:** Percy Guzman

**Affiliations:** Cancer Prevention Fellowship Program (CPFP), Division of Cancer Prevention (DCP), National Cancer Institute (NCI), Bethesda, Maryland, United States

**Keywords:** Emergency Services Utilization, Hospital Bed Management, Costa Rica Healthcare, Hospital Overcrowding, Triage and Bed Turnover

## Abstract

The emergency services in Costa Rica’s national hospitals are experiencing significant saturation, leading to increased patient observation times, extended hospital stays, and inefficient hospital bed management. This review examines national emergency service data from 2023, focusing on three major hospitals: *Hospital Rafael Ángel Calderón Guardia, Hospital San Juan de Dios*, and *Hospital México*. The analysis highlights the burden of non-urgent emergency visits, prolonged hospital stays, and the impact of high-burden conditions such as malignancies, circulatory diseases, and trauma on hospital bed turnover. International comparisons are presented, and recommendations for systemic improvements are proposed.

## 1. Introduction

Costa Rica’s healthcare system has consistently delivered high-quality medical services yet increasing emergency visits and hospital occupancy rates threaten its efficiency. In 2023, Costa Rica recorded 6,650,411 emergency visits, with three major hospitals handling 456,773 cases (6.87% of the national total). A significant portion of these cases were non-urgent, indicating inefficiencies in primary care and referral systems. This review aims to assess the key challenges in emergency service utilization and hospital bed management while comparing Costa Rica’s performance with international benchmarks.

## 2. Emergency Services Utilization in Costa Rica [1]

Among the three major hospitals, the number of emergency visits and their classification were as follows:

- Hospital Calderón Guardia: 98,330 emergency visits, with 71,665 (72.91%) classified as urgent.
- Hospital San Juan de Dios: 113,615 emergency visits, with 82,845 (72.93%) classified as urgent.
- Hospital México: 74,984 emergency visits, with 47,488 (63.37%) classified as urgent.

Hospital México recorded the highest proportion of non-urgent cases (36.67%), suggesting a higher misuse of emergency services. National data indicate that 52.34% of all emergency visits in Costa Rica were non-urgent, further exacerbating the overcrowding issue.

## 3. Impact of External Causes on Emergency Visits and Hospital Discharges

- External causes such as traffic accidents, falls, assaults, and machinery-related injuries accounted for 52.38% of all hospital discharges in 2023, despite representing only 2.66% of total emergency visits.
- This discrepancy suggests that while traumatic injuries impose a significant burden on hospital capacity, a considerable proportion of emergency visits arise from non-trauma-related conditions.

## 4. Hospital Stay Duration and Bed Turnover

The average hospital stay in Costa Rica in 2023 was 6.89 days, with notable variations across major hospitals:

- **Hospital Calderón Guardia:** 6.51 days;
- **Hospital San Juan de Dios:** 8.75 days;
- **Hospital México:** 7.79 days.

When compared internationally, Costa Rica’s hospital stays aligned with global trends but highlights areas for improvement. In Spain, the average hospital stay in 2021 was 8.3 days (7.9 days in public hospitals, 9.3 days in private facilities) [2]. In contrast, Mexico reported an average hospital stay of 5.7 days in hospitals implementing efficiency strategies [3]. The prolonged stays observed at Hospital San Juan de Dios and, to a lesser extent, Hospital México, indicate inefficiencies in hospital workflow, suggesting that improvements in discharge protocols, bed management, and patient flow optimization could reduce unnecessary hospital occupancy and enhance overall efficiency.

## 5. Impact of High Bed Occupancy on Turnover Rates

Costa Rica’s bed turnover rate in 2023 was 44.3, reflecting moderate efficiency. However, certain conditions significantly impact hospital bed availability due to their extended hospital stays:

- **Oncological conditions:** Hematopoietic cancers (15.46 days) and lung cancer (12.78 days) require prolonged hospitalization, limiting bed turnover.
- **Circulatory diseases:** Conditions such as acute myocardial infarction (8.15 days), hypertensive diseases (10.19 days), and other circulatory conditions (10.65 days) contribute to extended hospital occupancy.
- **Trauma-related admissions:** Fractures, external injuries, and other trauma-related cases demand hospital stays ranging between 7.37 and 9.26 days, delaying patient discharge and reducing overall hospital capacity.

## 6. Reduction in Hospital Bed Availability

Over the past four decades, Costa Rica has experienced a significant decline in hospital bed availability.

- In 1980, the country had 6926 hospital beds available, but by 2023, this number had dropped to 5374, representing a 22.4% reduction.
- This decline, coupled with increasing emergency visits and high bed occupancy rates, has exacerbated hospital congestion, making efficient bed turnover increasingly critical.

Costa Rica’s bed occupancy rate currently stands at 80.8%, reflecting sustained high utilization of hospital resources. Over the past two decades, while hospital bed capacity has declined, emergency visits have increased, straining inpatient services and delaying the discharge process. This imbalance has led to prolonged hospital stays and inefficient use of available resources.

When compared internationally, Costa Rica’s bed occupancy rate falls within the range of other healthcare systems:

- **United Kingdom (NHS):** ~85% [4].
- **Spain’s public hospitals:** 78% – 82% [2].
- **OECD average:** ~75% [5].
- **Germany:** ~77%.
- **Canada:** 75% – 85%, depending on the region [6].

These figures underscore the need for Costa Rica to optimize bed management strategies to align with international benchmarks and ensure efficient hospital resource utilization.

## 7. Implications and Recommendations

- **Reduce emergency room overuse:** Strengthen primary healthcare services and referral systems to address the high proportion of non-urgent cases.
- **Optimize hospital stays:** Implement discharge planning and post-hospitalization follow-up programs to enhance efficiency.
- **Improve bed management:** Reduce extended hospital stays for high-burden conditions to enhance bed turnover.
- **Enhance triage protocols:** Strengthen emergency department triage to prioritize urgent cases and redirect non-urgent visits.

## 8. Conclusion

Costa Rica’s emergency services face significant overcrowding, exacerbated by high rates of non-urgent visits and prolonged hospital stays due to high-burden conditions. Comparative analysis suggests that Costa Rica’s hospital efficiency can be improved by optimizing discharge processes and strengthening primary care systems. Addressing these challenges will enhance patient outcomes and ensure the sustainability of the healthcare system.
